# Innovative Clinical Pathways for Pregnant Women with Obesity: The Padua Project (Northeastern Italy)

**DOI:** 10.3390/jcm15176727

**Published:** 2026-08-30

**Authors:** Maria Grazia Dalfrà, Giovanni Sartore, Eugenio Ragazzi, Silvia Burlina, Silvia Visentin, Silvia Pastrolin, Marco Tosi, Erich Cosmi, Annunziata Lapolla

**Affiliations:** 1 DIMED-Diabetology and Dietetics Unit, University of Padova, 35128 Padova, Italy; mgdalfra@gmail.com (M.G.D.); g.sartore@unipd.it (G.S.); silvia.pastrolin@aulss6.veneto.it (S.P.);; 2 Studium Patavinum, University of Padova, 35122 Padova, Italy; eugenio.ragazzi@unipd.it; 3 Diabetes and Endocrinology Unit, ULSS8 Berica, 36071 Arzignano, Italy; silvia.burlina@aulss8.veneto.it; 4 DSDB-Obstetrics and Gynecology Clinic, University of Padova, 35128 Padova, Italy; silvia.visentin.1@unipd.it (S.V.); erich.cosmi@unipd.it (E.C.)

**Keywords:** gestational diabetes, obesity, pregnancy, adverse outcomes, preconception counseling

## Abstract

**Background/Objectives**: Maternal obesity is associated with an increased risk of adverse maternal and neonatal outcomes. This study evaluated whether participation in a multidisciplinary antenatal care pathway improved pregnancy outcomes compared with routine clinical practice in women with pre-pregnancy obesity. **Methods**: 711 women of reproductive age with preconception body mass index (BMI) ≥ 30 kg/m^2^ were identified from medical records collected between 2010 and 2023: 233 women were managed according to the multidisciplinary clinical pathway (pathway group), and 478 served as historical controls, followed at the same unit before pathway implementation. Age, BMI, gestational diabetes frequency, weight gain during pregnancy, gestational hypertension, preeclampsia/eclampsia, time and mode of delivery, birthweight, macrosomia, large for gestational age, neonatal hypoglycemia, hyperbilirubinemia, respiratory distress, fetal morbidity, and congenital anomalies were collected. Fasting plasma glucose, glycated hemoglobin, plasma glucose values under oral glucose tolerance test, plasma cholesterol and triglyceride levels were also evaluated. **Results**: The first evaluation occurred significantly earlier in the pathway group than in controls. Only about half of the women in the pathway group developed gestational diabetes mellitus (GDM) during pregnancy compared with 84% of controls (47% vs. 84%, *p* < 0.001), a difference partly explained by a referral bias in the control group, where women were referred after GDM screening rather than on the basis of obesity alone. As for maternal complications, the frequency of hypertension, eclampsia, and preeclampsia was similar in the two groups. As for fetal outcomes, pathway group women with respect to control showed a significantly lower frequency of preterm birth (5% vs. 11%, *p* = 0.0275), fetal morbidity (2% vs. 6%, *p* = 0.0377) and a tendency to lower frequency of macrosomia (7% vs. 12%, *p* = 0.0677), large for gestational age (19% vs. 26%, *p* = 0.116) and small for gestational age babies (5% vs. 7%, *p* = 0.50). **Conclusions**: This study shows that a multidisciplinary antenatal clinical approach was associated with a reduction in some adverse maternal–fetal outcomes related to pregnancy complicated by obesity, despite a less favorable baseline risk profile in the pathway group. Preconception counseling starting before pregnancy may represent an even more effective strategy, and efforts must be made in this direction.

## 1. Introduction

Obesity is the most prevalent noncommunicable disease worldwide. In US women of reproductive age (18–40 years), obesity rates have increased fourfold from 7.4% in 1976 to 27.5% in 2014; now >40% of women aged 20–39 years live with obesity [1]. Maternal obesity has been increasing in recent years all over the world, and globally there are about 39 million pregnancies per year complicated by maternal obesity [2]. In particular, in South Africa the prevalence of maternal obesity reaches 64%, in Mexico 65%, in the United States 55–63%, and in England the combined prevalence of overweight and obesity is 35% among 16–24-year-old women [3,4,5,6]. A recent meta-analysis estimated the current global prevalence of maternal obesity at 20.9% (95% CI, 18.6–23.1%) and projected that it will rise to 23.3% (95% CI, 20.3–26.2%) by 2030 [3]. It is important to emphasize that the highest frequency of pregnancies complicated by obesity is observed in areas of high deprivation in older mothers and in minority ethnic groups [6]. In Italy, data from the Istituto Superiore di Sanità related to the years 2016–2021 estimate a frequency of obesity in fertile women of 9% [7].

Obesity complicating pregnancy is a public health problem due to the fact that it is characterized by a series of maternal and fetal complications. A systematic review and meta-analysis clearly showed that gestational diabetes mellitus (GDM), preeclampsia, gestational hypertension, and cesarean delivery are more common among pregnant women with obesity [8]. In addition, maternal obesity has been associated with a higher risk of neonatal complications, including preterm birth, large-for-gestational-age (LGA) infants, congenital malformations, and perinatal death. Finally, evidence is now available showing that babies of pregnant women with obesity are at high risk of developing obesity, type 2 diabetes, and cardiovascular diseases later in life [9,10]. These complications determine increased costs mainly due to their treatment and duration of hospital stay [11]. So, preconception counseling, monitoring of weight gain, and strict follow-up during pregnancy are the keys to a safe pregnancy in women living with obesity planning pregnancy [12,13,14].

Despite the growing recognition of these risks, the evidence base on the optimal clinical management of pregnant women with obesity remains limited. Several national and international guidelines recommend a multidisciplinary approach to antenatal care in this population [12,13,14], but high-quality data on the real-world efficacy of structured multidisciplinary pathways are scarce. Most available studies have focused on specific isolated interventions, such as dietary counseling, physical activity programs, or GDM screening protocols, rather than on the impact of an integrated care model involving multiple professional figures working in a coordinated network. The few studies evaluating dedicated antenatal obesity clinics have generally been limited to women with severe obesity (body mass index, BMI, ≥40 kg/m^2^) [8], leaving a substantial gap in the evidence for women with class I and class II obesity, who represent the majority of pregnant women living with obesity in clinical practice. Furthermore, adherence to gestational weight gain recommendations and metabolic outcomes in real-world cohorts managed within structured pathways have rarely been reported in the literature, making it difficult to translate guideline recommendations into practical clinical models.

In our healthcare setting, we developed a multidisciplinary clinical pathway for the management of pregnant women with obesity based on an integrated network involving primary, secondary, and tertiary care providers [15]. Briefly, when a pregnant woman with obesity is evaluated for the first time by her gynecologist, an appointment is scheduled at the Pregnancy in Obesity Care Unit (POCU), a dedicated service within the Diabetology Unit. At POCU, an endocrinologist–diabetologist performs a comprehensive risk assessment and coordinates the patient’s follow-up according to protocols shared by the multidisciplinary team and in close collaboration with the referring gynecologist. Preliminary findings from the implementation of this pathway showed improved adherence to the Institute of Medicine (IOM) recommendations for gestational weight gain [15,16].

The present study extends the previously published feasibility analysis of the “Padua project” [15], a multidisciplinary clinical initiative developed at the Diabetology Unit of ULSS 6 Euganea, Padova, Italy, aimed at improving antenatal care for women with pre-pregnancy obesity through an integrated network of primary, secondary, and tertiary care providers. The present analysis evaluates maternal and neonatal outcomes over the first five years of full pathway implementation in a substantially larger cohort. Unlike the feasibility study, which focused primarily on process indicators and weight gain, the present analysis provides a comprehensive assessment of clinical outcomes across the full spectrum of obesity-related pregnancy complications, comparing women managed within the pathway with a historical control group of pregnant women with obesity followed at the same unit before pathway implementation. Accordingly, the aim of the present study was to investigate whether participation in this multidisciplinary clinical pathway was associated with improved maternal and neonatal outcomes during the first five years of its implementation compared with standard antenatal care in women with pre-pregnancy obesity.

## 2. Materials and Methods

### 2.1. Study Design

**Figure 1:**
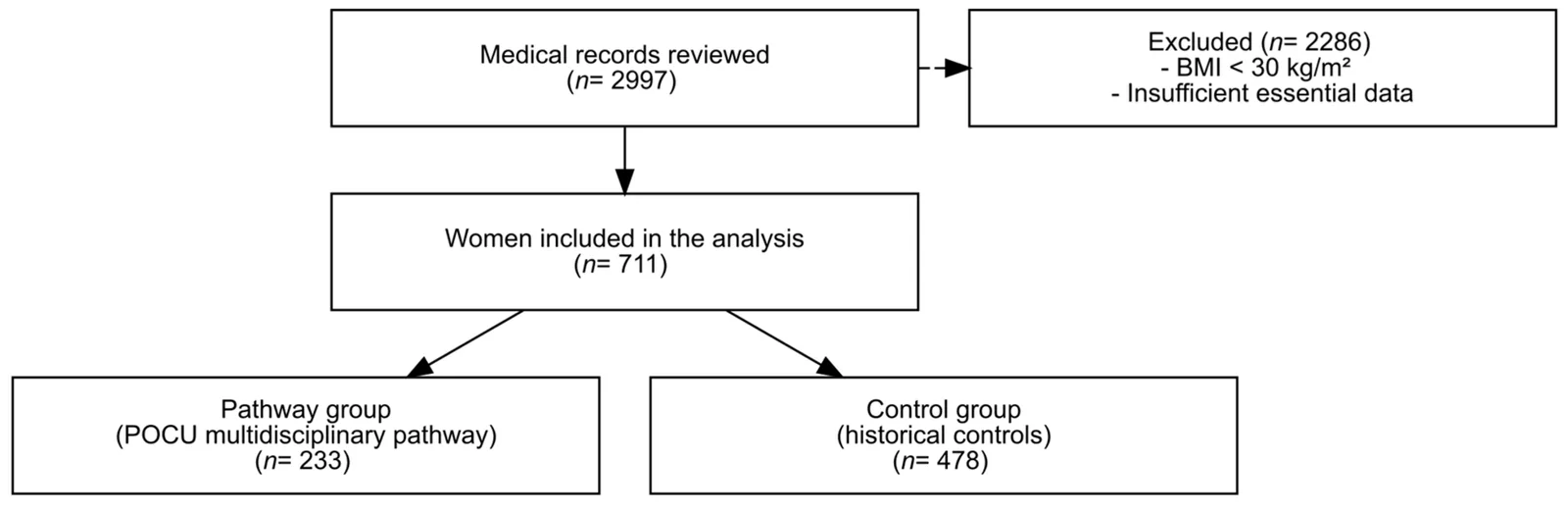
Flowchart of study participants.

We conducted a retrospective observational study with a historical control group, including pregnant women with pre-pregnancy obesity followed at the Diabetology Unit of UlSS 6 Euganea, Padova (Italy). The study included data from pregnant women followed at this unit between 2010 and 2023, and the multidisciplinary pathway was implemented in clinical practice from 2014. The clinical pathway was formally established under the institutional framework of Regione Veneto (DDR n. 25, 15 February 2018). Women managed according to the multidisciplinary clinical pathway of the Padua project were included in the pathway group, whereas pregnant women with pre-pregnancy obesity followed at the same unit starting before pathway implementation were used as historical controls. Maternal and neonatal outcomes were compared between the two groups. The flow of participants through the study is presented in Figure 1.

### 2.2. Participants

The study population consisted of women of reproductive age (range 18–46 years) with pre-pregnancy BMI ≥ 30 kg/m^2^, with no formal age exclusion criterion applied. Women in the pathway group were referred to the POCU on the basis of obesity alone, regardless of glucose status, whereas women in the control group were referred by community gynecologists after GDM screening, according to the clinical practice in place at that time. This difference in referral criteria between the two periods represents a relevant source of selection bias that must be considered when interpreting the GDM prevalence data.

At their first visit to the POCU, women undergo a comprehensive multidisciplinary assessment by an endocrinologist–diabetologist, a dietitian, a nurse, and an obstetrician from the High-Risk Pregnancy Service of the Department of Obstetrics and Gynecology at Padua University Hospital, which collaborates closely with the POCU.

The medical doctor, after a physical examination, counsels the woman on the maternal and fetal risks related to obesity in pregnancy.

The dietician provides nutritional advice to pregnant women with obesity in order to promote an adequate supply of nutrients to the mother and fetus and appropriate weight gain. Briefly, at the first nutritional visit, the expected weight gain during pregnancy is calculated using IOM guidelines [16]. The nutritional objectives are defined according to the body mass index, the weight gain, the dietary history, and the physical activity. The daily energy requirement is calculated taking into account the pre-pregnancy weight in order to guarantee an appropriate weight increase. During nutritional counseling at the initial visit, attention is focused on several important topics regarding the benefits of appropriate weight gain, nutrition, and exercise, such as limiting sugars and sugary drinks, composing a healthy and balanced dish, choosing low glycemic index foods and healthy snacks, drinking the right amount of water, have a daily 30 min walk. Furthermore, attention is paid to maintaining the carbohydrate amount above 175 g to ensure an adequate intake for both maternal and fetal brain requirements. Carbohydrates represent the main source of energy even during pregnancy; their contribution should be equal to 45-60% of total energy according to the Reference Intake Levels of Nutrients and Energy for the Italian Population (LARN) [17].

At each visit, weight and blood pressure are verified, and the nutritional advice is reviewed. Furthermore, at 16–18 and at 24–28 weeks of gestation (g.w.), the women are screened for GDM, as recommended by national and international guidelines; if the screening is negative, it has to be repeated at 24–26 g.w. [12,13,14,15,18]. If GDM is diagnosed, the women are included in the Pregnancy and GDM pathway managed at the unit.

During their first antenatal examination and periodically throughout the pregnancy, women are also evaluated by the obstetrician, who monitors fetal growth and well-being, and at term of pregnancy by the anesthesiologist to evaluate any difficulties during delivery [18].

The control group consisted of pregnant women with obesity who had been followed at the Diabetology Unit before the implementation of the multidisciplinary care pathway. These women were referred to the Diabetology Unit by community gynecologists after screening for GDM, according to the clinical practice in place at that time.

For all participants, the following maternal characteristics were collected: ethnicity, family history of type 2 diabetes, previous GDM, maternal age, pre-pregnancy body mass index (BMI, kg/m^2^), and BMI class (class I: 30.0–34.9 kg/m^2^; class II: 35.0–39.9 kg/m^2^; class III: ≥40.0 kg/m^2^). Maternal outcomes included the incidence of GDM (diagnosed according to the IADPSG criteria [19]), gestational weight gain, gestational hypertension, preeclampsia/eclampsia, gestational age at delivery, and mode of delivery. Neonatal outcomes included birth weight, macrosomia (defined as birth weight > 4 kg), large for gestational age (LGA, birth weight > 90th percentile), small for gestational age (SGA, birth weight < 10th percentile) based on standard Italian growth tables [20], neonatal hypoglycemia, hyperbilirubinemia, respiratory distress, and congenital malformations. Preterm birth was defined as delivery before 37 weeks of gestation.

Laboratory data included fasting plasma glucose, plasma glucose values during the oral glucose tolerance test (OGTT), total cholesterol, triglycerides, and glycated hemoglobin (HbA1c).

The study was conducted in accordance with the Declaration of Helsinki and its later amendments.

### 2.3. Analytical Determinations

Plasma glucose levels were measured using the glucose oxidase method [21]. Plasma lipids were measured as proposed by Dastych et al. [22]. HbA1c was measured by a standardized HPLC method aligned with IFCC [23].

### 2.4. Statistical Evaluation

Data were processed with JMP^®^ Version Pro 17 software for Windows (SAS Institute Inc, Cary, NC, USA). Continuous quantitative variables are expressed as means ± standard deviation (SD); categorical variables are expressed as absolute frequency or as percentages. Missing values in the database were treated with pairwise deletion in order to maximize all available data. Statistical significance was assessed by Student’s *t*-test for continuous data, and by Pearson’s chi-squared test for categorical data. A *p*-value < 0.05 was considered statistically significant. No correction for multiple comparisons was applied; results with *p*-values between 0.05 and 0.20 are therefore presented as hypothesis-generating trends and should not be interpreted as confirmed findings.

The feasibility of multivariable regression and propensity score matching was evaluated. A systematic assessment of missing data revealed that key variables had missing rates ranging from approximately 20% to over 40% in both groups, with high proportions observed for delivery characteristics (27–35%) and neonatal outcome variables (21–42%), as detailed in the Supplementary Materials (Table S1 and Figure S1). Under these conditions, complete-case multivariable analysis was considered feasible only for the parameters GDM and preterm delivery, which had the lowest proportions of missing data among the principal outcomes. For all other outcomes, complete-case analysis would have excluded a large and potentially non-random proportion of the study population and was therefore not performed. Multiple imputation was considered but judged not appropriate given that the missing data mechanism could not be verified in this retrospective dataset. Propensity score matching was also considered but was deemed methodologically inadequate in this specific context, as the two groups differ not only in baseline clinical characteristics but also in the time period of observation. Propensity score matching can balance measured covariates but cannot account for temporal confounding arising from changes in obstetric practice, screening protocols, and clinical management that occurred between the two observation periods. Multivariable logistic regression models were therefore fitted only for GDM and preterm delivery, including age, BMI, immigrant status, previous GDM, age over 35 years, and family history of type 2 diabetes as covariates. For all remaining outcomes, unadjusted comparisons are presented, with a full discussion of baseline imbalances and their potential influence on the results.

## 3. Results

A total of 2997 medical records were reviewed, and 711 women with a pre-pregnancy BMI ≥ 30 kg/m^2^ were identified and included in the analysis. Of these, 233 women were managed according to the multidisciplinary POCU clinical pathway (pathway group), whereas 478 women who had been followed at the same Diabetology Unit before pathway implementation served as controls. Baseline clinical and metabolic characteristics are summarized in Table 1. Compared with controls, women in the pathway group were younger, more frequently of immigrant origin, and had a higher pre-pregnancy BMI. The distribution of obesity classes also differed significantly between groups (*p* = 0.0007), with a lower proportion of class I obesity (52% vs. 67%) and a higher proportion of class II (35% vs. 24%) and class III obesity (13% vs. 9%) among women managed within the pathway. These findings indicate that the pathway group had a less favorable baseline risk profile than the control group.

Gestational weight gain was similar between groups across all obesity classes, with women in class III showing the lowest weight gain regardless of group assignment. Detailed values are reported in Table 1. The first evaluation occurred significantly earlier in women in the intervention group than in the control group (15.6 ± 6.2 vs. 20.6 ± 8.2 weeks of gestation, *p* < 0.0001). Moreover, only about half of the women in the pathway group developed GDM during pregnancy compared with 84% of those in the control group (47% vs. 84%, *p* < 0.0001; OR 0.16, 95% CI 0.11–0.23). Among women who developed GDM, only 14% in the pathway group required insulin therapy, compared with 39% in the control group (*p* < 0.05).

The mean values of total cholesterol, low-density lipoprotein (LDL) cholesterol, and triglycerides at diagnosis were significantly lower in the pathway group with respect to controls; no differences were found for high-density lipoprotein (HDL) cholesterol (Table 1).

As for maternal complications, the frequency of hypertension, eclampsia, and preeclampsia was similar in the two groups (Table 2).

Women in the pathway group showed a tendency toward a lower cesarean delivery rate with respect to the control ones (37% vs. 44%), but the difference was not statistically significant (*p* = 0.125); the frequency of preterm birth was significantly lower in the first group (5% vs. 11%, *p* = 0.0275; OR 0.40, 95% CI 0.15–0.95).

As regards the fetal outcomes (Table 3), the pathway group with respect to controls presented a tendency to a lower frequency of macrosomia, although not reaching statistical significance (7% vs. 12%, *p* = 0.0677); a similar trend was observed for the frequency of LGA and SGA babies, although it did not differ significantly between the two groups (LGA: 19% vs. 26%, *p* = 0.116; SGA: 5% vs. 7%, *p* = 0.50); conversely, fetal morbidity was higher in control group (2% vs. 6%, *p* = 0.0377; OR 0.29, 95% CI 0.05–1.01), mainly due to a higher frequency of hyperbilirubinemia (0.6% vs. 6%, *p* = 0.0094; OR 0.11, 95% CI 0.00–0.71).

To assess the robustness of the principal findings, multivariable logistic regression models were fitted for GDM and preterm delivery, including age, BMI, immigrant status, previous GDM, age over 35 years, and family history of type 2 diabetes as covariates. For GDM, the adjusted OR for pathway participation versus controls was 0.19 (95% CI 0.13–0.28, *n* = 693, 97.5% of the total sample), consistent with the unadjusted estimate. For preterm delivery, the adjusted OR was 0.33 (95% CI 0.12–0.79, *n* = 486, 68.4% of the total sample), also consistent with the unadjusted analysis. These findings suggest that the association between pathway participation and reduced GDM and preterm delivery is not explained by differences in baseline characteristics between groups. Adjusted analyses were not performed for fetal morbidity and macrosomia due to insufficient complete cases.

## 4. Discussion

The results of this study suggest that the implementation of a multidisciplinary clinical pathway for the management of pregnant women with obesity may contribute to reducing some adverse maternal and neonatal outcomes associated with this condition. In particular, compared with historical controls, women managed within the pathway experienced a significantly lower rate of preterm delivery and a trend toward a lower frequency of cesarean section. Regarding neonatal outcomes, a significant reduction in overall fetal morbidity was observed, mainly driven by a lower incidence of hyperbilirubinemia, together with trends toward lower rates of LGA, SGA, and macrosomia. Notably, these findings were observed despite the fact that women in the pathway group had a less favorable baseline risk profile, characterized by a higher pre-pregnancy BMI and a greater proportion of class II and class III obesity compared with controls. Although the observational design of the study precludes causal inference, these results are consistent with the hypothesis that an early, structured, and multidisciplinary approach may be associated with better management of pregnancies complicated by obesity and with more favorable maternal and neonatal outcomes.

Preterm birth is one important cause of fetal morbidity determined mainly by the prematurity of the newborn [24,25]. The global frequency of preterm birth is estimated to be between 5% and 18%, and its frequency is increasing in recent years [24]. The strong association of obesity and preterm delivery is demonstrated by a series of articles, as shown in a systematic review and meta-analysis [3]. Furthermore, in a paper by Sobczyk et al. [26], it has been shown that the frequency of preterm delivery in a cohort of 2794 pregnant women was 8% in normal weight and 10.3% in women with obesity, with an odds ratio for preterm delivery in women with obesity of 1.33 (95% CI: 0.98–1.79). The reduction in preterm delivery had a positive impact on fetal morbidity and, in particular, on hyperbilirubinemia, which is a consequence of early delivery [24,25]. Regarding the trend toward reduction, although not reaching significance, of the frequency of macrosomia, LGA and SGA babies, it is to notice that these negative outcomes often complicate the outcomes of the newborns of pregnant women with obesity; in particular, SGA could be a consequence of an inappropriate dietetic plan offered to these women [6,7,8,9,10].

Looking at the clinical characteristics of the patients under study, we must point out that the control group has a significantly higher mean age, a higher frequency of development of GDM, and a worse lipid profile compared to the pathway group, and this could partially explain the higher maternal and fetal morbidity compared to the cases. On the other hand, the women in the pathway group were more frequently immigrants with respect to controls, so at higher risk of developing negative outcomes during pregnancy due to frequently low income [5,6].

Another relevant finding of the study is that women enrolled in the pathway were evaluated significantly earlier in gestation than controls (15.6 vs. 20.6 weeks), allowing close metabolic surveillance from the first trimester onwards. This early assessment was associated with a lower frequency of GDM and a lower need for insulin therapy among women diagnosed with GDM (14% vs. 39%), which is clinically relevant given that insulin requirement during pregnancy is associated with a higher risk of adverse maternal and neonatal outcomes. The GDM prevalence observed in both groups is considerably higher than the 10–14% typically reported in the general Italian pregnant population and also higher than the 30–50% range commonly cited for pregnant women living with obesity in the international literature. This reflects the specific nature of our clinical setting, as our unit functions as a tertiary referral center for metabolic disorders in pregnancy, and both groups represent a highly selected population in which pre-pregnancy obesity coexists with multiple additional GDM risk factors, including family history of type 2 diabetes, advanced maternal age, high-risk ethnicity, and history of previous GDM. The difference in GDM prevalence between the two groups is further explained by a difference in referral criteria between the two periods: women in the control group were referred by community gynecologists specifically after GDM screening, which means that women with abnormal glucose metabolism were systematically over-represented in that cohort. Women in the pathway group, by contrast, were referred on the basis of obesity alone regardless of glucose status, which partially dilutes the observed GDM prevalence. Both groups were diagnosed according to the IADPSG criteria, ensuring diagnostic consistency across the two periods.

Gestational weight gain was within the range recommended by the Institute of Medicine (IOM) for pregnant women with obesity [16] in both study groups, indicating adequate weight management. In this context, the optimal gestational weight gain for women with obesity remains a matter of debate. A study evaluating energy expenditure during pregnancy demonstrated that appropriate weight gain can be achieved when daily energy intake does not exceed total energy expenditure. The authors suggested that the additional energy required for fetal growth should be largely provided through the mobilization of maternal fat stores rather than by increasing caloric intake. Based on these findings, they proposed a daily energy deficit of approximately 100 kcal as the most appropriate strategy to achieve the recommended gestational weight gain in women with obesity [27]. Moreover, recent studies have shown that a minor or no increase in weight or modest weight loss can reduce the risk of LGA without a significant increase in SGA, suggesting that the IOM recommendations should be re-evaluated and the weight gain diversified by obesity class [28,29,30].

The need for close follow-up by a multidisciplinary team is underlined by numerous guidelines [12,13,14,18,31], but very scarce data are reported in the literature on the real efficacy of this approach. Denison et al. [32] have demonstrated that a multidisciplinary approach similar to ours is useful to reduce the frequency of stillbirths and SGA and improve the detection of GDM; however, their approach is dedicated to pregnant women with BMI ≥ 40 kg/m^2^ [11,32]. Our data extend some positive results to women with BMI ≥ 30 kg/m^2^ too; therefore, despite the multidisciplinary intervention, maternal and fetal outcomes did not improve as we expected. The multicenter prospective study DALI (vitamin D and lifestyle intervention in the prevention of gestational diabetes mellitus) has evaluated whether lifestyle intervention or vitamin D supplementation is able to reduce GDM frequency in pregnant women with obesity. The results of the study show that the lifestyle intervention was not able to reduce GDM in the second trimester but limited gestational weight gain and reduced the frequency of LGA babies, while no other beneficial effects were reported [33]. A series of aspects can affect pregnancy outcome in women living with obesity; lifestyle changes could be difficult to obtain with the increase in gestational weeks, and psychological aspects can contribute to this difficulty, so probably other strategies in addition to those employed must be implemented.

This study has several strengths. First, it allowed the evaluation of a wide range of clinically relevant maternal and neonatal outcomes, providing a comprehensive assessment of the impact of the multidisciplinary care pathway in routine clinical practice. Second, the availability of a comparison group consisting of women with obesity followed at the same Diabetology Unit before the implementation of the pathway enabled the assessment of outcomes within a similar healthcare setting, while minimizing variability related to differences in clinical organization. Finally, the relatively large sample size represents a further strength, increasing the robustness of the findings.

Some limitations must be considered carefully when interpreting these results. The retrospective observational design does not allow causal conclusions to be drawn, and all findings should be interpreted as associations. The use of a historical control group is the most important structural limitation of the study, as it introduces potential temporal bias: changes in obstetric practice, GDM screening protocols, neonatal care standards, and referral patterns that occurred between the two observation periods may have contributed independently to the observed differences in outcomes. However, it should be noted that the nutritional counseling protocols and educational content were substantially consistent between the two periods, as the same national guidelines (IOM, LARN) were applied throughout the observation period at this unit.

The baseline differences between groups represent a further limitation. Women in the pathway group were younger, had a higher pre-pregnancy BMI, a greater proportion of class II and III obesity, a lower prevalence of previous GDM, a different ethnic composition, and were evaluated approximately five weeks earlier than controls, as discussed in detail above. The absence of adjusted analyses for most outcomes arises from the substantial proportion of missing data in key variables, as detailed in the Supplementary Materials and in the Section 2.4. Multivariable logistic regression was feasible only for GDM and preterm delivery; for all remaining outcomes, the extent of missing data precluded adjusted analyses. Information on adherence to the multidisciplinary intervention, including visit attendance, compliance with nutritional counseling, and uptake of physical activity recommendations, was not systematically recorded and could not be included in the analysis. Similarly, data on comorbidities associated with obesity in reproductive-age women, including polycystic ovary syndrome and metabolic syndrome, were not comprehensively available across both groups. Finally, the single-center design, the high proportion of immigrant women, and the predominance of higher obesity classes limit the generalizability of these findings to other healthcare systems, ethnic populations, and women with class I obesity. Prospective multicenter studies with standardized data collection and concurrent control groups will be necessary to confirm these findings and to identify which components of the multidisciplinary pathway contribute most to improved outcomes.

## 5. Conclusions

This study shows that a structured multidisciplinary antenatal care pathway, including early referral, dedicated nutritional counseling, and close metabolic monitoring, was associated with a significant reduction in preterm delivery and overall fetal morbidity in women with pre-pregnancy obesity, despite a less favorable baseline risk profile in the pathway group. These findings support the value of an integrated multidisciplinary approach to the management of obesity during pregnancy and contribute real-world evidence to a field where high-quality implementation data remain scarce.

Preconception counseling aimed at achieving weight reduction and promoting healthy lifestyle behaviors before conception may represent an even more effective strategy for reducing obesity-related pregnancy complications [34]. Our center has developed a dedicated preconception clinical pathway based on an integrated network of primary, secondary, and tertiary care providers, but preliminary data showed a very low uptake of this service, accounting for only 2% of all appointments within the network [15]. Increasing awareness and accessibility of preconception care should therefore be considered a priority. Prospective multicenter studies with standardized protocols and concurrent control groups are needed to confirm these findings and to determine which components of the multidisciplinary pathway contribute most to improved maternal and neonatal outcomes.

## Figures and Tables

**Table 1:** Clinical and metabolic characteristics of the study population.

Parameters	Pathway Group	Control Group	*p* ^a^
	(*n* = 233)	(*n* = 478)	
Age, y	31.4 ± 5.0	33.7 ± 4.9	<0.0001
BMI, kg/m^2^	35.35 ± 3.96	34.38 ± 3.98	0.0023
BMI class (I/II/III), %	52/35/13	67/24/9	0.0007
Immigrant, %	55	40	<0.0001
Parity, %	67/32/1	64/35/1	0.6696
Family-based risk, %	51	46	0.2787
Age > 35 y risk, %	24	38	0.0002
Previous GDM, %	6	26	<0.0001
Weight gain, kg	7.8 ± 5.9	7.7 ± 6.2	0.8817 (0.818 ^b^)
Weight gain for class I obesity, kg	8.6 ± 5.7 (*n* = 99 ^c^)	8.5 ± 5.8 (*n* = 253 ^c^)	0.94
Weight gain for class II obesity, kg	8.3 ± 5.9 (*n* = 66 ^c^)	9.9 ± 6.3 (*n* = 89 ^c^)	0.1637
Weight gain for class III obesity, kg	4.1 ± 5.7 (*n* = 28 ^c^)	4.2 ± 6.9 (*n* = 35 ^c^)	0.9211
Week of 1st visit, wk	15.6 ± 6.2	20.6 ± 8.2	<0.0001
1st FPG, mg/dL	88.2 ± 9.9	92.3 ± 13.6	<0.0001
GDM diagnosis, %	47	84	<0.0001
Weeks of 1st lipid profile, wk	20.8 ± 5.6	23.7 ± 5.4	<0.0001
Total cholesterol at diagnosis, mg/dL	211 ± 42	228 ± 46	<0.0001
HDL cholesterol at diagnosis, mg/dL	66 ± 16	66 ± 14	0.9926
LDL cholesterol at diagnosis, mg/dL	114 ± 34	126 ± 40	0.0005
Triglycerides at diagnosis, mg/dL	155 ± 63	182 ± 66	<0.0001

^a^ Statistical significance was assessed by Student’s *t*-test for continuous data, and by Pearson’s chi-squared test for categorical data. ^b^ Corrected for delivery week. ^c^ *n* refers to the number of women with available data after pairwise deletion. Parity is presented as nulliparity/low multiparity (parity 1 to 3)/grand multiparity (parity 4 to 6). Abbreviations: HDL: high-density lipoprotein; LDL: low-density lipoprotein; FPG: fasting plasma glucose.

**Table 2:** Maternal outcomes of the study population.

Parameters	Pathway Group (*n* = 233)	Control Group (*n* = 478)	*p* ^a^
Gestational hypertension, %	13	11	0.4291
Eclampsia, %	0.4	0.7	0.7143
Pre-eclampsia, %	0.9	0.9	0.9880
Insulin therapy, %	14	39	0.057
Delivery gestational week	39.0 ± 2.1	38.4 ± 1.9	0.0019
Delivery (spont/induc/cesar/abort), %	40/21/37/2	34/21/44/1	0.1887
Preterm delivery, %	5	11	0.0275

^a^ Statistical significance was assessed by Student’s *t*-test for continuous data, and by Pearson’s chi-squared test for categorical data. Abbreviations, with respect to delivery: spont: spontaneous; induc: induced; cesar: cesarean section; abort: abortion.

**Table 3:** Fetal outcomes.

Parameters	Pathway group (*n* = 233)	Control group (*n* = 478)	*p* ^a^
Birth weight, kg	3392 ± 496	3375 ± 566	0.7630
AGA/LGA/SGA, %	76/19/5	67/26/7	0.1935
Macrosomia, weight > 4000 g, %	7	12	0.0677
Hypoglycemia, %	1	1	0.9667
Hyperbilirubinemia, %	0.65	6	0.0094
Respiratory distress (yes/no), %	1	3	0.3449
Fetal morbidity ^b^ (yes/no), %	2	6	0.0377
Fetal malformations (yes/no), %	0.7	2	0.2658

^a^ Statistical significance was assessed by Student’s *t*-test for continuous data, and by Pearson’s chi-squared test for categorical data. ^b^ Fetal morbidity: birthweight, macrosomia, LGA and SGA, neonatal hypoglycemia, hyperbilirubinemia, respiratory distress, and fetal malformations were considered. Abbreviations: AGA: appropriate for gestational age; LGA: large for gestational age; SGA: small for gestational age.

## Data Availability

The raw data supporting the conclusions of this article will be made available by the authors on request.

## Supplementary Materials

The following supporting information can be downloaded at https://www.mdpi.com/article/10.3390/jcm15176727/s1: Table S1: Missing data analysis for variables included in Table 1, Table 2 and Table 3, by group (pathway group, *n* = 233; control group, *n* = 478). Values are expressed as absolute numbers and percentages of missing observations. Figure S1: Heatmap of missing data for variables included in Table 1, Table 2 and Table 3. Patients are ordered by group (control, *n* = 478; pathway group, *n* = 233). Black cells indicate missing values; grey cells indicate available data. Overall missing rate: 20.4%.

## References

[1] Schon S.B., Cabre H.E., Redman L.M. (2024). The impact of obesity on reproductive health and metabolism in reproductive-age females. Fertil. Steril..

[2] Chen C., Xu X., Yan Y. (2018). Estimated global overweight and obesity burden in pregnant women based on panel data model. PLoS ONE.

[3] Kent L., McGirr M., Eastwood K.A. (2024). Global trends in prevalence of maternal overweight and obesity: A systematic review and meta-analysis of routinely collected data retrospective cohorts. Int. J. Popul. Data Sci..

[4] NHS Statistics on Obesity, Physical Activity and Diet, England—2020. https://www.gov.uk/government/statistics/statistics-on-obesity-physical-activity-and-diet-england-2020-ns.

[5] Wells J.C.K., Marphatia A.A., Cole T.J., McCoy D. (2012). Associations of economic and gender inequality with global obesity prevalence: Understanding the female excess. Soc. Sci. Med..

[6] Heslehurst N., Sattar N., Rajasingam D., Wilkinson J., Summerbell C.D., Rankin J. (2012). Existing maternal obesity guidelines may increase inequalities between ethnic groups: A national epidemiological study of 502,474 births in England. BMC Pregnancy Childbirth.

[7] Masocco M., Minardi V., Contoli B., Minelli G., Manno V., Cobellis L., Greco D. (2023). Sovrappeso e obesità nella popolazione adulta in Italia. Trend temporali, differenze socio-anagrafiche e regionali con focus sulla regione Campania. Boll. Epidemiol. Naz..

[8] Mukhtar F. (2025). A Systematic Review of the Management of Maternal Obesity in Pregnancy: Antenatal Management, Outcomes, and Long-Term Implications on Maternal Health. Cureus.

[9] Tan H.C., Roberts J., Catov J., Krishnamurthy R., Shypailo R., Bacha F. (2015). Mother’s pre-pregnancy BMI is an important determinant of adverse cardiometabolic risk in childhood. Pediatr. Diabetes.

[10] Eriksson J.G., Sandboge S., Salonen M.K., Kajantie E., Osmond C. (2014). Long-term consequences of maternal overweight in pregnancy on offspring later health: Findings from the Helsinki Birth Cohort Study. Ann. Med..

[11] Denison F.C., Norwood P., Bhattacharya S., Duffy A., Mahmood T., Morris C., Raja E.A., Norman J.E., Lee A.J., Scotland G. (2014). Association between maternal body mass index during pregnancy, short-term morbidity, and increased health service costs: A population-based study. BJOG.

[12] Modder J., Fitzsimons K.J. (2010). Management of Women with Obesity in Pregnancy.

[13] American College of Obstetricians and Gynecologists (2013). Committee Opinion No. 549: Obesity in pregnancy. Obstet. Gynecol..

[14] Lapolla A., Dalfrà M.G., Sbraccia P., Nisoli E., Vettor R. (2016). Clinical management of overweight and obesity. Recommendations of the Italian Society of Obesity 2016. Obesity in Pregnancy.

[15] Lapolla A., Scibetta D., Gallina P., Iorizzo G., Dalfrà M.G., Visentin S., Nardelli G.B., Vettor R. (2018). Innovative clinical pathways for obese pregnant women: Design and feasibility of the Padua project (North-Eastern Italy). J. Endocrinol. Investig..

[16] Rasmussen K.M., Yaktine A.L., Institute of Medicine, National Research Council (2009). Weight Gain During Pregnancy: Reexamining the Guidelines.

[17] Società Italiana di Nutrizione Umana (SINU) (2023). LARN. Livelli di Assunzione di Riferimento di Nutrienti ed Energia per la Popolazione Italiana.

[18] AGENAS Linee di Indirizzo Clinico-Organizzative per la Prevenzione delle Complicanze Legate alla Gravidanza. http://www.agenas.it.

[19] International Association of Diabetes and Pregnancy Study Groups Consensus Panel (2010). International association of diabetes and pregnancy study groups recommendations on the diagnosis and classification of hyperglycemia in pregnancy. Diabetes Care.

[20] Bertino E., Spada E., Occhi L., Coscia A., Giuliani F., Gagliardi L., Gilli G., Bona G., Fabris C., De Curtis M. (2010). Neonatal anthropometric charts: The Italian neonatal study compared with other European studies. J. Pediatr. Gastroenterol. Nutr..

[21] Huggett A.S., Nixon D.A. (1957). Use of glucose oxidase, peroxidase, and O-dianisidine in determination of blood and urinary glucose. Lancet.

[22] Dastych M., Wiewiofka O., Benovska M. (2014). Ethamsylate (Dicynone) interference in determination of serum creatinine; uric acid; triglycerides and cholesterol in assays involving the Trinder reaction: In vivo and in vitro. Clin. Lab..

[23] Mosca A., Paleari R., Dalfrà M.G., Di Cianni G., Cuccuru I., Pellegrini G. (2006). Reference intervals for hemoglobin A1c in pregnant women: Data from an Italian multicenter study. Clin. Chem..

[24] Ohuma E.O., Moller A.B., Bradley E., Chakwera S., Hussain-Alkhateeb L., Lewin A., Okwaraji Y.B., Mahanani W.R., Johansson E.W., Lavin T. (2023). National, regional, and global estimates of preterm birth in 2020, with trends from 2010: A systematic analysis. Lancet.

[25] The Lancet (2016). The unfinished agenda of preterm births. Lancet.

[26] Sobczyk K., Holecki T., Woźniak-Holecka J., Grajek M. (2022). Does Maternal Obesity Affect Preterm Birth? Documentary Cohort Study of Preterm in Firstborns-Silesia (Poland). Children.

[27] Most J., Amant M.S., Hsia D.S., Altazan A.D., Thomas D.M., Gilmore L.A., Vallo P.M., Beyl R.A., Ravussin E., Redman L.M. (2019). Evidence-based recommendations for energy intake in pregnant women with obesity. J. Clin. Invest..

[28] Robillard P.Y., Dekker G., Boukerrou M., Le Moullec N., Hulsey T.C. (2018). Relationship between pre-pregnancy maternal BMI and optimal weight gain in singleton pregnancies. Heliyon.

[29] Devlieger R., Ameye L., Nuyts T., Goemaes R., Bogaerts A. (2020). Reappraisal of Gestational Weight Gain Recommendations in Obese Pregnant Women: A Population-Based Study of 337,590 Births. Obes. Facts.

[30] Faucher M.A., Barger M.K. (2015). Gestational weight gain in obese women by class of obesity and select maternal/newborn outcomes: A systematic review. Women Birth.

[31] Fitzsimons K.J., Modder J. (2010). Setting maternity care standards for women with obesity in pregnancy. Semin. Fetal Neonatal Med..

[32] Denison F.C., MacGregor H., Stirrat L.I., Stevenson K., Norman J.E., Reynolds R.M. (2017). Does attendance at a specialist antenatal clinic improve clinical outcomes in women with class III obesity compared with standard care? A retrospective case-note analysis. BMJ Open.

[33] Harreiter J., Desoye G., van Poppel M.N.M., Kautzky-Willer A., Dunne F., Corcoy R., Devlieger R., Simmons D., DALI Consortium, Adelantado J.M. (2019). The Effects of Lifestyle and/or Vitamin D Supplementation Interventions on Pregnancy Outcomes: What Have We Learned from the DALI Studies?. Curr. Diab. Rep..

[34] Dalfrà M.G., Burlina S., Lapolla A. (2022). Weight gain during pregnancy: A narrative review on the recent evidences. Diabetes Res. Clin. Pract..

