# Supplementary material for: Innovative Clinical Pathways for Pregnant Women with Obesity: The Padua Project (Northeastern Italy)
Source: J Clin Med. 2026 Aug 30;15(17):6727. doi: 10.3390/jcm15176727 (PMC13565772; doi:10.3390/jcm15176727)

**Table S1**. Missing data analysis for variables included in Tables 1–3, by group (pathway group, *n* = 233; control group, *n* = 478). Values are expressed as absolute number and percentage of missing observations.

| Table | Variable | Pathway group | | | Control group | | |
|---|---|---|---|---|---|---|---|
| | | *n* | *n* missing | % missing | *n* | *n* missing | % missing |
| Table 1 - Baseline characteristics | Age (years) | 233 | 0 | 0 | 478 | 0 | 0 |
| Table 1 - Baseline characteristics | BMI (kg/mÂ²) | 233 | 0 | 0 | 478 | 0 | 0 |
| Table 1 - Baseline characteristics | BMI class | 233 | 0 | 0 | 478 | 0 | 0 |
| Table 1 - Baseline characteristics | Ethnic origin | 233 | 22 | 9.4 | 478 | 108 | 22.6 |
| Table 1 - Baseline characteristics | Family history of T2D | 233 | 0 | 0 | 478 | 3 | 0.6 |
| Table 1 - Baseline characteristics | Age > 35 years | 233 | 0 | 0 | 478 | 0 | 0 |
| Table 1 - Baseline characteristics | Previous GDM | 233 | 2 | 0.9 | 478 | 2 | 0.4 |
| Table 1 - Baseline characteristics | Gestational weight gain (kg) | 233 | 40 | 17.2 | 478 | 101 | 21.1 |
| Table 1 - Baseline characteristics | Week of first visit | 233 | 64 | 27.5 | 478 | 146 | 30.5 |
| Table 1 - Baseline characteristics | Fasting plasma glucose (mg/dL) | 233 | 22 | 9.4 | 478 | 41 | 8.6 |
| Table 1 - Baseline characteristics | GDM diagnosis | 233 | 6 | 2.6 | 478 | 7 | 1.5 |
| Table 1 - Baseline characteristics | Week of lipid profile assessment | 233 | 140 | 60.1 | 478 | 328 | 68.6 |
| Table 1 - Baseline characteristics | Total cholesterol at diagnosis (mg/dL) | 233 | 40 | 17.2 | 478 | 125 | 26.2 |
| Table 1 - Baseline characteristics | HDL cholesterol at diagnosis (mg/dL) | 233 | 41 | 17.6 | 478 | 133 | 27.8 |
| Table 1 - Baseline characteristics | LDL cholesterol at diagnosis (mg/dL) | 233 | 46 | 19.7 | 478 | 137 | 28.7 |
| Table 1 - Baseline characteristics | Triglycerides at diagnosis (mg/dL) | 233 | 41 | 17.6 | 478 | 129 | 27 |
| Table 2 - Maternal outcomes | Gestational hypertension | 233 | 1 | 0.4 | 478 | 19 | 4 |
| Table 2 - Maternal outcomes | Eclampsia | 233 | 1 | 0.4 | 478 | 20 | 4.2 |
| Table 2 - Maternal outcomes | Pre-eclampsia | 233 | 1 | 0.4 | 478 | 20 | 4.2 |
| Table 2 - Maternal outcomes | Insulin therapy | 233 | 3 | 1.3 | 478 | 20 | 4.2 |
| Table 2 - Maternal outcomes | Gestational week at delivery | 233 | 81 | 34.8 | 478 | 142 | 29.7 |
| Table 2 - Maternal outcomes | Mode of delivery | 233 | 75 | 32.2 | 478 | 130 | 27.2 |
| Table 2 - Maternal outcomes | Preterm delivery | 233 | 81 | 34.8 | 478 | 142 | 29.7 |
| Table 3 - Neonatal outcomes | Birth weight (kg) | 233 | 81 | 34.8 | 478 | 170 | 35.6 |
| Table 3 - Neonatal outcomes | AGA/LGA/SGA classification | 233 | 88 | 37.8 | 478 | 199 | 41.6 |

| Table | Variable | Pathway group | | | Control group | | |
|---|---|---|---|---|---|---|---|
| | | *n* | *n* missing | % missing | *n* | *n* missing | % missing |
| Table 3 - Neonatal outcomes | Macrosomia (birth weight > 4000 g) | 233 | 50 | 21.5 | 478 | 169 | 35.4 |
| Table 3 - Neonatal outcomes | Neonatal hypoglycemia | 233 | 77 | 33 | 478 | 177 | 37 |
| Table 3 - Neonatal outcomes | Hyperbilirubinemia | 233 | 78 | 33.5 | 478 | 177 | 37 |
| Table 3 - Neonatal outcomes | Respiratory distress | 233 | 78 | 33.5 | 478 | 177 | 37 |
| Table 3 - Neonatal outcomes | Fetal morbidity | 233 | 77 | 33 | 478 | 177 | 37 |
| Table 3 - Neonatal outcomes | Congenital malformations | 233 | 78 | 33.5 | 478 | 178 | 37.2 |

**Figure S1**. Heatmap of missing data for variables included in Tables 1–3. Patients are ordered by group (control, *n* = 478; pathway group, *n* = 233). Black cells indicate missing values; grey cells indicate available data. Overall missing rate: 20.4%.

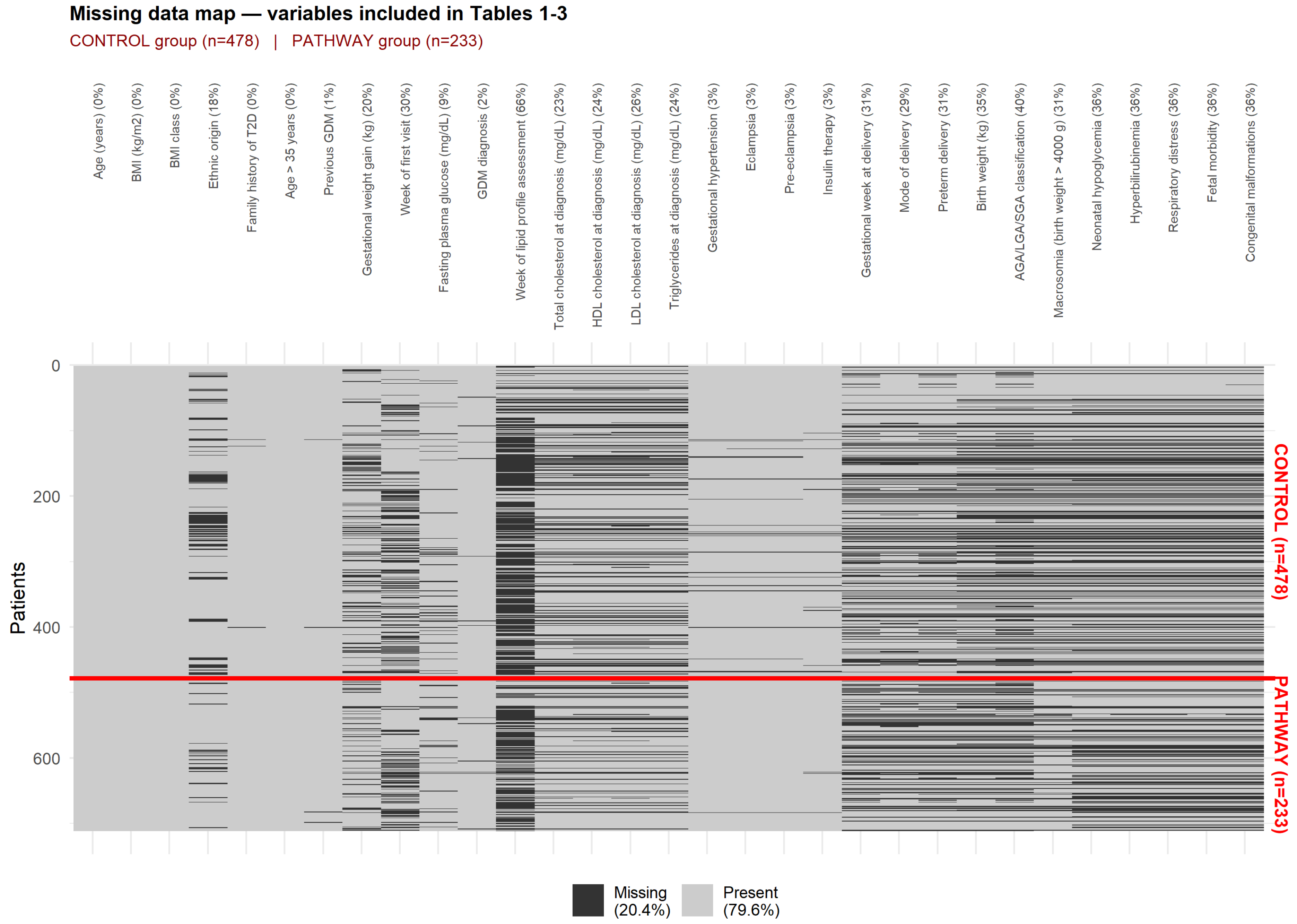

Supplement: Supplementary file 1 [file jcm-15-06727-s001.zip › jcm-4504875-supplementary.pdf]
